# Factors affecting the implementation of childhood vaccination communication strategies in Nigeria: a qualitative study

**DOI:** 10.1186/s12889-017-4020-6

**Published:** 2017-02-15

**Authors:** Afiong Oku, Angela Oyo-Ita, Claire Glenton, Atle Fretheim, Glory Eteng, Heather Ames, Artur Muloliwa, Jessica Kaufman, Sophie Hill, Julie Cliff, Yuri Cartier, Xavier Bosch-Capblanch, Gabriel Rada, Simon Lewin

**Affiliations:** 1 Community Medicine Department, University of Calabar, P.M.B 1115, Calabar Municipality, Cross River State Nigeria; 2 Norwegian Institute of Public Health, Postboks 4404 Nydalen, 0403 Oslo, Norway; 3 Institute of Health and Society, University of Oslo, P.O box 1130, Blindern, 0318 Oslo Norway; 4 Sociology Department, University of Calabar, P.M.B 1115, Calabar Municipality, Cross River State Nigeria; 5 Departamento de Saúde, Direcção Provincial de Saúde de Nampula, Av. SamoraMachel n° 1016 R/C, C.P. N° 14, Nampula, Mozambique; 6 Centre for Health Communication and Participation, School of Psychology and Public Health, La Trobe University, Health Sciences 2, Victoria, 3086 Australia; 7 Faculdade de Medicina, Universidade Eduardo Mondlane, Maputo, Mozambique; 8 International Union for Health Promotion and Education, 42 Blvd. de la Libération, 95203 St. Denis, Cedex France; 9 Swiss Tropical and Public Health Institute, Socinstrasse 57, 4051 Basel, Switzerland; 10 University of Basel, Petersplatz 1, 4003 Basel, Switzerland; 11 Evidence-based Healthcare Program, Pontificia Universidad Católica de Chile, Avda. Libertador Bernardo O’Higgins 340, Santiago, Chile; 12 Health Systems Research Unit, South African Medical Research Council, Francie van Zijl Drive, Parowvallei, PO Box 19070, 7505 Tygerberg, South Africa

**Keywords:** Communication strategies, Vaccination, Nigeria, Barriers, Facilitators, Qualitative study

## Abstract

**Background:**

The role of health communication in vaccination programmes cannot be overemphasized: it has contributed significantly to creating and sustaining demand for vaccination services and improving vaccination coverage. In Nigeria, numerous communication approaches have been deployed but these interventions are not without challenges. We therefore aimed to explore factors affecting the delivery of vaccination communication in Nigeria.

**Methods:**

We used a qualitative approach and conducted the study in two states: Bauchi and Cross River States in northern and southern Nigeria respectively. We identified factors affecting the implementation of communication interventions through interviews with relevant stakeholders involved in vaccination communication in the health services. We also reviewed relevant documents. Data generated were transcribed verbatim and analysed using thematic analysis.

**Results:**

We used the SURE framework to organise the identified factors (barriers and facilitators) affecting vaccination communication delivery. We then grouped these into health systems and community level factors. Some of the commonly reported health system barriers amongst stakeholders interviewed included: funding constraints, human resource factors (health worker shortages, training deficiencies, poor attitude of health workers and vaccination teams), inadequate infrastructure and equipment and weak political will. Community level factors included the attitudes of community stakeholders and of parents and caregivers. We also identified factors that appeared to facilitate communication activities. These included political support, engagement of traditional and religious institutions and the use of organised communication committees.

**Conclusions:**

Communication activities are a crucial element of immunization programmes. It is therefore important for policy makers and programme managers to understand the barriers and facilitators affecting the delivery of vaccination communication so as to be able to implement communication interventions more effectively.

**Electronic supplementary material:**

The online version of this article (doi:10.1186/s12889-017-4020-6) contains supplementary material, which is available to authorized users.

## Background

Globally, vaccination is recognized as a cost-effective public health measure for decreasing childhood mortality and morbidity [1]. Strategies which improve the uptake of vaccination include ‘supply-side’ interventions, such as ensuring a constant supply of potent vaccines, strong health systems to ensure delivery of these vaccines and sufficient health personnel to administer vaccines [2]; and ‘demand-side’ components which focus on individual and household determinants of health-seeking behaviours, such as building the knowledge base of individuals to utilise vaccination programmes to their advantage. Addressing vaccine hesitancy linked to parental knowledge, understanding, attitudes, beliefs, and behaviours is an important example of a demand-side component [3–9].

Poor communication, if not addressed, can undermine several components of vaccination delivery, including vaccine acceptance [3]. Improving vaccination communication delivery is therefore crucial to achieving better vaccination outcomes [10, 11] as well as the greater goal of knowledgeable caregivers and communities – important contributors to improving child health in many settings [12–14]. Effective communication could improve uptake of childhood vaccination, address incomplete vaccination or missed children, further strengthen routine immunization programmes, and encourage the use of new and underused vaccines. Although communication is an invaluable tool in routine and campaign childhood vaccination activities, as well as in other health programmes, it is rarely addressed in a systematic way compared with other components of vaccination programmes [3]. Ideally, vaccination communication efforts should complement and boost other immunization components, such as service provision, quality of care, capacity-building and the skills of health personnel, and disease notification and surveillance [15].

In Nigeria, where this study was based, routine vaccination coverage for all recommended vaccines has remained poor though there has been a gradual rise in vaccination coverage from 21% of eligible children (0–11 months of age) in 2003 to 25% a decade later [16]. Factors seen to have contributed to poor routine immunization performance include ineffective supply chains, poor delivery of services, scarce human resources, low demand for health services, funding gaps, accountability issues and weak governance, and poor data quality [17]. Furthermore, vaccine hesitancy – defined as “a delay in the acceptance or refusal of vaccines despite the availability of vaccine services” [18] – may also play an important role. Vaccine-hesitant individuals are a mixed group: individuals may delay receiving vaccines, or may agree to vaccines but be unsure of doing so, or may decline some vaccines but agree to others, as commonly observed in some parts of northern Nigeria in the context of oral polio vaccine mass campaigns [13]. For example, studies have shown that the increased number of polio campaigns in Nigeria were seen as suspicious by some populations [19, 20].

Communication interventions have made significant contributions to the polio eradication programme in Nigeria [21]. Numerous communication interventions have been implemented, particularly in high-risk states for polio, with the aim of increasing acceptance of routine immunization and breaking the transmission of wild poliovirus. However, implementing these communication interventions has been challenging. This paper aims to explore factors affecting the delivery of vaccination communication in Nigeria. An understanding of such factors can inform policy makers during the planning of communication interventions and when adapting these to suit local contexts. This study forms part of the ‘Communicate to vaccinate’ (COMMVAC) research project which focuses on building research evidence to improve communication about childhood vaccinations with parents, caregivers and communities in LMICs. In this study, communication interventions refer to all interventions which are purposeful, structured, repeatable and adaptable strategies aimed at informing and influencing individual and community decisions on personal and public health participation, disease prevention and promotion, policy making, service improvement and research [12, 22].

## Methods

### Study setting

The setting for the study was Nigeria, the fourteenth largest country by landmass in Africa, with a projected population of over 180 million people in 2016. Nigeria is divided administratively into 36 States and the Federal Capital Territory (FCT) Abuja. Each State is further divided into Local Government Areas (LGAs), which are made up of several wards. The Nigerian people have diverse cultures, religions and ethnicities.

In Nigeria, the agency responsible for controlling vaccine-preventable diseases through the provision of vaccines and immunization guidelines is the National Primary Health Care Development Agency (NPHCDA). National responsibility for the development of communication interventions for vaccination programmes is given to the National Social Mobilization Working Group, while State and Local Social Mobilization Committees are responsible for coordination and implementation of communication interventions at the State and Local Government levels. The routine immunization schedule in Nigeria recommends that all childhood vaccinations are completed by nine months of age. In addition to routine immunization, numerous rounds of mass campaigns are also held in all the states of the country as part of efforts to eradicate poliomyelitis. Campaigns are also carried out occasionally for meningococcal meningitis, measles and yellow fever vaccines.

### Study sites

We conducted the study in rural and urban settings of Bauchi and Cross River States in northern and southern Nigeria. We also conducted interviews with national-level decision makers in Abuja, the capital city. We selected Bauchi and Cross River States based on differences in vaccination coverage rates, with lower rates in Bauchi compared to Cross River (DPT3 coverage rates of 12.5 and 76.1% respectively) [16]; and differences in terms of vaccine hesitancy, with vaccine refusal being more common in Bauchi, related to religious and cultural beliefs [23]. Bauchi was selected over Borno and Yobe States in northern Nigeria, which recorded the lowest vaccination rates in the country, as security issues in those two states made research difficult. In addition, at the time that the study was conducted in 2014, Bauchi was among the 12 polio prevalent states of northern Nigeria and has received both global and national attention to eliminate polio and improve vaccination uptake, with appreciable resources directed to vaccination communication activities. Cross River State was selected to provide an example of a good performer in terms of vaccination coverage, with vaccination coverage second only to Rivers State (52.5 and 55.5% respectively) [16]. Cross River has received less national and international attention than Bauchi and has maintained polio-free status for over a decade. Lastly, the religious settings of the two states are different: Bauchi is predominantly Muslim while Cross River is predominantly Christian. These differences in religious beliefs may impact on beliefs about and attitudes towards vaccination.

### Study design

The study used a qualitative approach, based on data from key informant interviews.

### Sampling

We purposively selected stakeholders involved in vaccination activities and who played active roles in the planning or implementation of childhood vaccination communication strategies at different levels of health care delivery, and who had the potential to provide rich, relevant and diverse data pertinent to the study objective. These stakeholders included policy makers, programme managers, social mobilization officers/health educators and representatives from relevant organizations including UNICEF, the World Health Organization and the Vaccine Alliance (GAVI). We conducted a total of 15 interviews (Table 1).

**Table 1:** List of stakeholders interviewed

Level	Respondent	Total
National	Senior communication staff at UNICEF, WHO, GAVI and the National Polio Emergency Centre	4
State	Social Mobilization Officer (State Health Educator) (two in Cross River and one in Bauchi)	3
	Deputy Director, Community Health Services (Bauchi)	1
	State Immunization officer (one in Cross River and one in Bauchi)	2
	Deputy Director, Immunization Services (Bauchi)	1
Local	Local Immunization Officer (Bauchi/Cross River)	2
	Local Social Mobilization Officer (Bauchi/Cross River)	2
Total		15

### Data collection methods

Data collection took place from January to April 2014. We used a semi-structured interview guide (Additional file 1) to gain insights into the factors affecting the implementation of communication interventions in Nigeria. The interview team comprised the principal investigator (AO) as moderator and a note taker who took down notes of both verbal and non-verbal responses. The interviews were carried out at a convenient time and place chosen by the respondent, were conducted in English, and lasted 30–45 min on average. We recorded each interview session once informed consent had been sought and obtained. At the end of each interview, we transcribed the recorded sessions verbatim and placed them in a file bearing the date the interview was conducted, the place and the research questions that the interview addressed. We tried to ensure anonymity as far as possible but because many respondents held very senior positions, it was difficult to ensure complete anonymity. All data files were securely stored.

### Data analysis

Two researchers (AO and GB) carried out data analysis using a framework thematic analysis approach [24, 25] which involved four steps: familiarization, indexing/coding, charting and mapping/interpretation. First, we familiarized ourselves with the data collected by listening repeatedly to the audio recordings and studying the transcripts. This helped us gain an overview of the body of material gathered and to become aware of key ideas as well as recurrent themes. Our next step was to identify portions of the data that corresponded to a particular theme (indexing or coding). To enhance the validity of the coding, the principal investigator (AO) and a sociologist (GB) from the University of Calabar coded the data and each developed a coding book. We then coded each interview transcript independently and later merged our findings. We went through each interview transcript and extracted information on possible factors affecting the implementation of childhood vaccination communication interventions. The SURE (Supporting the Use of Research Evidence) Framework, a theory-informed conceptual framework, offered us a useful starting point for our analysis as it provided us with a comprehensive list of possible factors that could influence the successful implementation of interventions [26]. We identified a number of themes when looking through the data, which we then organised under different categories and sub-categories, drawing on the SURE Framework (Additional file 2). Financial constraints, health resources, inadequate infrastructure and equipment were grouped in the ‘health system constraints’ domain; issues related to politics were grouped in the ‘social and political’ domain; while community level factors brought together the SURE framework domains of ‘recipients of care’ and ‘providers of care’.

Thereafter, we lifted the indexed data from its original textual context and put these data in charts that organized the themes into categories and sub-categories. Interesting data extracts and central themes were used verbatim to illustrate key findings. As themes emerged these were indexed and compared with themes from subsequent interviews. Lastly, we did a mapping and interpretation which involved the analysis of the key characteristics as laid out in the charts.

## Results

The factors we identified were grouped into three sub-categories: health system level factors, political factors and community level factors.

### Health system factors

#### Financial constraints

In both states, all respondents interviewed at the different levels of the health care system consistently mentioned that inadequate funding was the main barrier to the implementation of vaccination communication interventions. However, this concern was expressed more strongly in Cross River than in Bauchi. While one of the respondents confirmed that poor funding had been found to disrupt all aspects of the vaccination programme, he also pointed out that communication is usually worse hit, with the smallest allocation, or often nothing, for routine immunization. The respondents reported that funding gaps led to poorly implemented communication activities in terms of coverage and frequency of messaging.

Most respondents noted that communication interventions for mass campaigns generally receive significantly more funding and resources than routine immunization programmes. This gap was attributed by most respondents to the absence of donor or partner involvement in communication activities, especially for routine immunization activities, which donors viewed as the responsibility of the government. Some respondents pointed out that communication activities around routine immunization were particularly limited in frequency and range. This, they suggested, was due to the fact that communication activities were not specifically budgeted for in routine immunization programmes, which was evident from an absence of communication interventions between campaigns. Some respondents, however, argued that communication activities were generally underfunded for both campaigns and routine immunization activities and were never given priority attention when immunization programmes were planned.

A national stakeholder commented:

“*In October 2012, when we had a campaign for meningitis, Nigeria’s communication budget for that campaign was only two percent of the total budget for the campaign and yet we expect miracles to happen. Same way, if you look at the communication budget in other programmes, I’m sure you will be shocked to see that communication always receives the least budget. So, if this does not change – because one thing in communication is that what you give in is what you get out and communication is not something you do once and you stop.”* (Decision maker, national level)

Campaigns only received the desired attention among the public, they stressed, if communication was on the priority list for funding by development partners and donor agencies. The overdependence of states and local governments on the federal government to fund communication activities also further contributed to the problem.

The effects of the funding gap for vaccination communication were felt most at the local government and state levels with responsibility for delivering communication interventions, and played out in two ways. Firstly, some respondents at the state level reported late release of funds for communication activities. This was observed to delay disbursement of needed materials (printed posters and other information, education and communication materials), especially to hard-to-reach areas.

Secondly, at local government levels, the local mobilizers confirmed that funding gaps contributed to delays in implementing activities and sometimes to a failure to implement these activities at all. Respondents cited instances when materials produced for particular campaigns arrived at the local government late, at the end of the campaign, or not at all. This occurred because funds were not readily available to transport these materials to the local government areas. In some cases, materials remained in storage at the state level and were not distributed to local government areas.

Respondents also described accountability issues, with funds earmarked for communication activities occasionally being diverted to address other pressing needs, further delaying the implementation of communication activities, particularly as local funding was difficult to access. This was more commonly reported in Cross River than in Bauchi. Funding constraints were sometimes cushioned by development partners who provided funds for specific activities during campaigns in some local government areas. Health workers, especially those at the local government level, tried to solve the issues related to funding delays by using their personal monies and private vehicles to meet their targets for monthly routine activities in their respective local government areas:

*“In terms of funding, especially for campaigns, funds are provided but it is never enough for our planned mobilization activities. This affects the range of activities one performs. Most times, we have to use our own funds to succeed. If you want a wider coverage, they may give you funds for a specific number but you may go out of your way to reach more people.”* (Mobilization officer, Cross River State)

*“I use my salary now to do my activities, especially for routine immunization activities, to meet up and be able to present my report at the State meeting.”* (Local social mobilization officer, Bauchi)

#### Inadequate infrastructure and equipment

Some respondents at the state and local levels highlighted the fact that the basic requirements to conduct an effective and extensive community mobilization for vaccination were not readily available. The state mobilization unit, which coordinates communication activities at the lower levels, lacked well-equipped offices, computers, vehicles and motorcycles or other means of transportation. Other equipment needed for these activities were not readily available such as megaphones, public address systems used for announcements and printed communication materials such as flyers, leaflets and posters:

*“A lack of mobility is a major challenge. You definitely cannot carry out an effective social mobilization work without mobility because you need to cut across many places. We do not have any vehicles attached to this department.”* (Mobilization officer, Cross River State)

#### Human resource factors

##### Health worker shortages

Most respondents at the state and local levels in both states referred to the general shortage of health personnel, especially in rural areas where more than 70% of the population reside. This deficit in human resources affected the delivery of vaccination communication and the immunization programme as a whole. Health worker shortages affected rural areas particularly, where some health facilities had only one health worker responsible for the various tasks in the vaccination clinic such as registration of clients, conducting health education sessions and administration of vaccines.

*“Every health worker wants to work in the urban areas, especially those whose husbands are politicians, and every big man wants his wife to be in the urban area. So when you transfer them it’s a big problem. This has resulted in many of them in the urban and very few health workers in the rural areas.”* (Immunization Officer, Cross River State)

Respondents in Bauchi noted that this gap was partly addressed through the use of volunteer community mobilizers, and the use of traditional and religious leaders as community mobilizers.

##### Training deficiencies

While some respondents alluded to the fact that there was an organised structure to manage communication activities at the national, state and local levels, other stakeholders pointed out that the structure on the ground did not translate into having qualified personnel at the community level to meet the objectives of the immunization programme. They highlighted the lack of well-trained communication personnel as a barrier to the effective mobilization of communities, especially the lack of personnel at local levels. They observed that even after training, personnel at the local level may not be able to meet the desired objectives of the programme effectively because of a lack of proper supervision and monitoring at this level. One of the respondents noted that failure to see the need to train and prepare these health workers to effectively deliver vaccination communication messages was related to the fact that they were under the responsibility of the local government, not the State Ministry of Health. This meant that the state had no control over the local government health workers and did not see these health workers as their responsibility. He further explained that the local government usually depended on the State Ministry of Health to supervise and monitor the health workers at the lower levels, but this rarely happened. One respondent was also of the view that health workers had poor communication and negotiation skills and were not able to communicate the purpose of their visit well, especially when they visited resistant households. This, the respondent noted, may have contributed to their poor performance in the field.

Another respondent described how the ‘cascade’ model of training was partly responsible for the training gap. He explained that before a campaign, state social mobilization officers or health educators are trained directly by the national level to deliver training to the local Social Mobilization officers. The local Social Mobilizer is then expected to train “unqualified community members” at the ward level. The respondent described how dilution occurred, with the quality of the training declining at each stage, leading to poor training outcomes:

*“You will find out, especially at the local government level, that the local Social Mobilizers who are saddled with communication assignments are not trained communication personnel. So you end up training and training and training. Some people are not just trainable*.” (Development partner)

##### Poor attitudes of health workers and vaccination teams

Poor attitudes among health workers at the state and local government levels and a lack of commitment to social mobilization activities outside campaigns were also reported to impact negatively on communication interventions for vaccination programmes. For instance, in situations where there was a delay in funding at the national level, respondents reported that mobilizers would not begin mobilizing the communities in which they worked but would instead wait for the funds to be disbursed before initiating mobilization activities. This was said to have led to poor performance by these vaccination teams in terms of achieving vaccination coverage outcomes. The reasons behind this may be health workers’ previous experiences of not being paid or being underpaid for services rendered or having to use their own resources to conduct communication activities, with reimbursement often being delayed. Respondents also stressed that many of the vaccinators were not committed to meeting the objectives of the programme but were instead interested in what they stood to gain financially.

### Political factors

Most respondents viewed the presence of political support as a major facilitator while the absence of political support was seen to undermine the delivery of health interventions. They noted that communication interventions for routine immunization would be more likely to achieve their objectives if they were given similar levels of political support to that given to campaigns. Political support for mass campaigns varied across states and local governments and tended to be stronger in high-risk states or local government areas which had national or international attention or where political leaders were given mandates to improve their vaccination coverage, as we describe in more detail below.

#### Failure of state and local governments to own the vaccination programmes

Some development partners involved in the implementation of communication interventions noted that most state and local political leaders failed to show ownership of the immunization programme. This was more of a problem in the southern states compared to the North, which enjoyed more donor support. The development partners noted that political leaders failed to provide funds to carry out communication interventions in their states or local government areas or failed to disburse these funds in a timely manner or to train and deploy health staff and provide the materials and equipment needed to effectively deliver routine vaccination services. The reason given by some respondents included an over-dependence on development partners and the fact that political leaders are usually more interested in committing their resources to more visible infrastructure, such as roads and schools. One of the partners reported that only a few states he had worked in had demonstrated ownership of programmes and taken the lead in providing the necessary funds and making decisions:

*“If the states and the local government own this programme, you don’t need money from partners. For instance, a state that owns a radio station, a television station, you see all these stations should have been running free jingles. But they never do that. If you want to run anything, they ask you to pay even if it is their own children and mothers that will benefit. If there is ownership, those things will not happen, it is only once in a while in some States during campaigns you see them giving those orders. Immediately after the campaign, announcement stops.”* (Development partner)

*“At the national level the states are asked to develop their communication interventions and thereafter they don’t have money to implement, and expect funds from the national to implement this and that hardly happens except for the funding that UNICEF sends because UNICEF is the mandate agency for polio communication.”* (Development Partner)

*“We, the partners, are running after the government in some states whereas in other states, even when you go there and take over the driver’s seat, it is still almost impossible to drive the government to follow you and you can’t be at the forefront of any programme because the communities will still not see you as one of theirs. They hardly ever have enough funds to implement communications activities in the context of health programmes*.” (Development Partner)

#### Health communication interventions not a priority among policy makers

Most respondents reported that while most policy makers were inattentive towards health issues in general, this also applied to health communication which was viewed as a sort of “optional add-on” to the array of health workers’ tasks. Respondents argued that health communication, whether for routine or mass campaigns, was usually perceived as a minor service component and was not seen as important or necessary. One of the factors contributing to this problem seemed to be the assumption by policy makers that health care workers do not require any training in communication skills but do require more training on technical components of the immunization programme. Some respondents reported that the level of funding allocated to communication activities demonstrated the perceived lack of priority, as discussed above. This attitude towards communication activities, they explained, trickles down to all levels of government and results in health communication not being given the attention it needs, as one of the respondents noted:

*“In our national budgeting, health is not one of those areas that attract funding. Even when immunization is considered, the funds allotted are usually for other technical components and communication is rarely considered and this trickles down to the local government level. Because if the national government does not allocate adequate resources to health or communication, the states will not see any reason to do that, so this now boils down to who is in charge. If the person in charge does not have an interest in health, then health is treated as unimportant*.” (Development Partner)

### Community level factors

#### Attitudes of community stakeholders

Respondents also discussed the attitudes of community stakeholders in certain communities. One respondent reported that in some rural areas, community members demand money from health workers in exchange for immunization services even when they understand the benefits of the programme. This, they suggested, was because community members believed that health workers were paid well to bring the services to them. In some instances, community gatekeepers were also reported as having prevented campaigns from being organised in their setting and having insisted that government provide basic necessities such as accessible roads, schools and health services before these campaigns could take place. This occurred rarely in some communities but was more often seen in hard-to-reach areas where people felt marginalized:

*If you go to the community now they believe you came with money to give them. If you don’t give them they will sabotage your activities so that is why we have problems. Because you need people to come and get immunization sometimes we have to give them some incentives before they help in delivery of vaccination messages. I had an experience in the past when you go into the community, they say to you, “You people are enjoying yourselves under air-condition and driving big cars and yet we are suffering”. So once you give them something they cooperate with you and take the messages to their communities*.” (Senior Health officer, Bauchi)

#### Attitudes among community members

Similar issues were raised by respondents across the two states. As expected, vaccine resistance was more frequently referred to by respondents in Bauchi than in Cross River State and respondents reported that this tended to affect negatively the reception of communication messages. In Bauchi, this resistance was particularly seen in response to polio campaigns. One reason given by respondents was the large number of polio campaigns which they suspected had led community members to believe that the government was concentrating its resources on polio while neglecting their felt needs. In addition, certain religious groups and anti-polio vaccine campaigners have spread rumours about the inclusion into the vaccine of anti-fertility drugs or the HIV virus, as way of checking population growth in Muslims. In Cross River, respondents reported that pockets of resistance existed among certain religious groups in some communities.

*“Refusals of polio vaccine still persist in some communities. There is a video tape being circulated by one Muslim teacher discouraging people against vaccination which led to a lot of rejections of the polio vaccine and resulted in our vaccination teams embarking on house-to-house immunization to be attacked because of the tape”* (Local Social Mobilizer, Bauchi)

*“People are very hesitant when it comes to immunization campaigns, and have a phobia for polio campaigns in this part of the country. That is why our most important problem is this one. Even when people have heard about the campaigns through radio messages and are aware of it, they are still sceptical about immunization campaigns generally. People accept routine immunization but the campaign is what they are rejecting. They believe they go to the clinic and come back, but for the campaign why do we then come to their houses? They get suspicious and think there is more to it than we are telling them, which is why they reject it. But for the routine they go to the hospital and clinic. They don’t have any query or complain about it.”* (Senior Health officer, Bauchi)

#### Engagement of traditional and religious institutions

The engagement of traditional and religious institutions was seen to facilitate the delivery of communication for childhood vaccination in both states, and particularly in Bauchi where resistant families and communities were commonly found. All respondents indicated that such engagement was a major boost to the immunization programme since these institutions were trusted and respected in many communities. This intervention was seen to improve the demand for vaccination and to counter resistance in certain religious groups and communities.

The cooperation and support of traditional and religious leaders as advocates for immunization played a significant role in the vaccination programme, particularly in delivery of announcements in their churches or mosques and being part of community dialogue teams to tackle the problem of vaccine hesitancy in certain households and communities.

#### Organisation of communication committees

At the national level, some respondents noted that the presence of a national Social Mobilization Working Group that comprised of multiple developmental partners and highly skilled personnel was a major plus to the delivery of vaccination communication. This group developed the strategic plan for communication and trained health personnel at the state level. Similarly, in the states and local government areas, the State and Local Social Mobilization Committees were described as useful in coordinating and engaging appropriate channels for vaccination communication. In certain local government areas, the presence of a functional Ward Development Committee (a committee that provides links between the community and the health system) was seen to contribute significantly in executing communication activities at the community level.

## Discussion

Communication has been described as a core component of service delivery in the immunization programme and can play an important role in ensuring that children are fully vaccinated [22, 27–29]. However, our study suggests that vaccination communication was poorly understood by policy makers, with little mention of capacity building in communication or communication in the wider context of social mobilization.

Our study identified a number of other factors that were reported as influencing the successful implementation of vaccination communication strategies for both routine immunization and mass campaigns. Weak political commitment impacted negatively on communication strategies for routine immunization services and contributed to difficulties with funding, deployment and training of staff, and provision of equipment and transportation especially at lower levels of the health system. Indeed, funding was a major challenge in the implementation of most components of immunization delivery in both states. This was confirmed in the Comprehensive EPI multi-year plan where communication and advocacy received the least budgetary allocation compared to other components [30], and is consistent with the results from recent studies conducted in Cameroon and Nigeria [31, 32]. Poor funding played a significant role in many of the barriers identified in this study.

Several studies have suggested that regular exposure through mass media and community channels is key to promoting vaccination [33–35], although the evidence on the effects of such community-aimed interventions to inform and educate about childhood vaccination is still quite weak [36]. Furthermore, a lack of communication activities outside campaigns may result in people not recalling vaccination messages about routine immunization. The implication of this is that if messages are not given continuously people may forget or may not attach importance to the issue.

In many settings, health workers are seen as the most important sources of information for parents deciding whether their child should receive a vaccine [37, 38]. A lack of health workers, especially in rural and hard-to-reach communities, has important impacts on the effective delivery of communication interventions. Additionally, the absence of skilled communication personnel, especially at lower levels of the health system, may limit the capacity to counter negative information about vaccines and achieve community support for vaccination programmes [21], as observed in this study. The training of health workers needs to strongly address interpersonal communication skills, so that health workers can maximize on any opportunities for reinforcement on immunization and child health more generally. Such training can help to ensure that health workers provide relevant and comprehensible information in a respectful and culturally sensitive manner [27].

As also noted in other studies [20, 39], the engagement and cooperation of traditional and religious leaders was seen to facilitate the delivery of communication interventions for childhood vaccination in Nigeria, particularly in the context of campaigns, and to contribute to meeting the immunization programme’s objectives. The engagement of traditional and religious institutions was more intensive in Bauchi compared to Cross River, as rejection of the oral polio vaccine was seen as a major challenge in the former. In Bauchi State, resistance was targeted mainly towards polio campaigns following rumours and misconceptions that the vaccine included anti-fertility drugs or the HIV virus, as an indirect method of checking population growth in the predominantly Muslim states in the North. These rumours are similar to those reported in other studies [18–21]. Engaging religious and traditional leaders has also been described as a useful and acceptable intervention in other countries with large Muslim communities [40, 41]. Such interventions may be helpful in addressing communities’ concerns about vaccination and the vaccination process, although their impacts need to be evaluated [42].

In Table 2, we provide suggestions on areas where health systems needed to be strengthened. Respondents suggested that policy makers might consider improving the funding allocation for communication activities and introducing regular vaccination messages outside campaigns. They also suggested that systems be established for the management and timely disbursement of funds within vaccination communication programmes, especially at the local level. This could ensure adequate planning and timely implementation of communication activities for childhood immunization. Accountability systems also need to be put in place and integrated into systems that work to ensure that immunization funds are released and used efficiently. We suggest that vaccination programme managers and other decision makers need to consider strategies to ensure that parents and caregivers in rural and hard-to-reach communities have access to information on childhood vaccination. This could include providing information through routes other than health workers as well as strategies to improve the retention and quality of health workers in these areas. Lastly, we suggest that the training of health workers be purposeful and enable health workers to target communication to different groups in communities. Putting in place a system to monitor the appropriateness and quality of training activities at the local level should be considered while training needs assessments should be conducted from time to time.

**Table 2:** Where health systems need to be strengthened in relation to communication for childhood vaccination

Health system issue	Key findings from the analysis	Implications for the strengthening of the health system to support vaccination communication
Funding of vaccination communication interventions	• Least budgetary allocation to communication and social mobilization • Funds/incentives seldom available for routine immunization and some costs borne by health workers • Overdependence on donors • Problems and delays with disbursement of funds and materials at lower levels of the health system • Lack of funding for sustained communication programmes for routine immunization • Communication strategies intermittent (minimal between campaigns)	• Consider improving the funding allocation to communication activities, which should be continuous even after campaigns • Provide a regular source of funding for routine immunization communication activities in the recurrent budget of States and Local Government Areas as this may improve sustainability • Ensure that systems are available for the management and timely disbursement of funds within vaccination communication programmes, especially at the local level
Equipment and transportation	• Lack of equipment (information, education and communication (IEC) materials, megaphones and vehicles) for adequate mobilization • Transportation difficulties	• State and local government Social Mobilization Committees and Health Promotion Departments should be strengthened to develop their own IEC materials
Human resources for health	• Generally seen as inadequate • Inequities in distribution of human resources, with more resources in the urban than in rural Local Government Areas	• Consider redistribution of health workers, temporary staff from the pool of retirees or community volunteers who can serve as mobilizers • Consider providing incentives for health workers in rural settings
Training	• Lack of human resources for supervision of frontline health care providers • Training deficiencies, with large numbers of communication personnel not sufficiently skilled • ‘Cascade’ training model results in dilution of training efforts	• Establish a system to monitor the appropriateness and quality of training activities at the local level • Training needs assessments should be conducted from time to time • Supervision of Local Government Area mobilizers by state social mobilisers should be strengthened • Frontline communicators in the various Local Government Areas should be provided with training guides or manuals which can be tailored to meet local needs
Health provider attitudes	• Health providers, including vaccination teams, poorly motivated	• Ensure that vaccination teams are provided with incentives
Attitudes of parents and caregivers towards vaccination	• Vaccine hesitancy and rejection in some religious groups may impede receipt of vaccination information	• Engagement of traditional and religious institutions and other community structures may be useful in countering refusal in some communities
Political support	• Political support focused on campaigns only • Failure of State and Local Governments to take ownership of programmes • Health communication not seen as a priority by some policy makers • Lack of political commitment in some Local Government Areas	• Regular advocacy visits to political leaders • Improve accountability systems, particularly at the state and local government level, to prevent misappropriation of funds meant for the communication needs of the vaccination programme
Community participation	• Lack of community participation	• Consider evidence–informed and locally appropriate interventions to involve communities in planning and implementation of communication intervention for both routine immunization and campaigns

### Strengths and limitations of the study

The main strength of the study was the iterative and flexible nature of the qualitative research approach that we adopted when conducting the interviews. We had the opportunity to go back to the respondents for clarification of certain issues and to ask questions which were not adequately addressed in earlier interviews. We also looked at the national, state and local levels of health care delivery in the country, allowing a more complete picture of vaccination communication issues to be obtained.

A potential limitation is that the study was conducted during the pre-eradication era of polio in Nigeria, when the attention of governments and international agencies was focused primarily on polio eradication. This may have skewed our findings towards issues relevant to communication in the context of campaigns. In addition, we did not interview senior staff in the National Directorate of Disease Control and Immunization responsible for routine immunization programme activities. However, we included immunization officers at both state and local levels of health care delivery and it is likely that these respondents will have covered similar ground to those at the national level.

## Conclusion

Our earlier work has shown that a wide range of communication interventions are being used to promote uptake of childhood vaccination in Nigeria [32]. However, a number of health system factors such as funding constraints, inadequate infrastructure and equipment, health worker-related and political factors as well as community level factors, such as the attitudes of community stakeholders and members, were found to hinder the delivery of vaccination communication interventions. Important differences were observed across and within the two states studied. Most of the barriers to implementing vaccination communication strategies found in this study were more strongly expressed in Cross River State, and also in rural compared to urban areas. These differences can be attributed to differences in infrastructure, resources (human and financial) and accountability as a consequence of investments in the polio eradication programme in Bauchi State.

Programme managers and front line providers reported that the most consistent barrier to delivering vaccination communication was inadequate funding. This, they suggested, has greatly impacted on vaccination communication delivery and the disbursement of communication materials, especially to areas where they are most needed. In resource constrained settings like those studied, systems should be put in place to improve efficiency in how available resources are utilized. For instance, gains could be made by integrating routine EPI messaging into vaccination campaigns or packaging this with communication around other well-funded childhood interventions. Another important barrier was the absence of strong political will at Federal and Local government levels for implementing communication strategies for routine immunization. This could be attributed to a poor understanding among political leaders of the importance of vaccination communication within the routine immunization programme.

Decision makers need to look at how to address these barriers so as to facilitate the implementation at scale of evidence-informed strategies for communicating with parents and caregivers regarding childhood immunization. Addressing communication gaps, especially in routine immunization services, will require bridging the current funding gap, addressing human resource deficits and ensuring strong political will for implementation. Facilitators for implementation of vaccination communication interventions, such as the engagement of traditional and religious institutions and the use of organised communication committees, should be strengthened. If sufficiently planned, funded, and integrated with service delivery, vaccination communication activities could meet their desired objectives.

## Additional files

Additional file 1: Guide for interviews with programme managers, social mobilization officers and development partners. (PDF 340 kb)
Additional file 2: SURE Framework of key domains for the identification of factors affecting the implementation of policy options. (PDF 248 kb)

## Abbreviations

COMMVAC 2: ‘Communicate to Vaccinate’ Project 2
DPT3: Combined diptheria, pertusis and tetanus vaccine, three doses
EPI: Expanded programme on immunization
FCT: Federal Capital Territory
GAVI: The Vaccine Alliance
LGA: Local Government Area
LMIC: Low and middle income countries
NPHCDA: National Primary Health Care Development Agency
WHO: World Health Organization
