# Supplementary material for: Factors affecting the implementation of childhood vaccination communication strategies in Nigeria: a qualitative study
Source: BMC Public Health. 2017 Feb 15;17:200. doi: 10.1186/s12889-017-4020-6 (PMC5311723; doi:10.1186/s12889-017-4020-6)
Supplement: Additional file 1: — Guide for interviews with programme managers, social mobilization officers and development partners. (PDF 340 kb) [file 12889_2017_4020_MOESM1_ESM.pdf]

Additional file 1: Guide for interviews with programme managers, social mobilisation officers and development partners

Your participation in this interview is totally voluntary. Do you have any questions before we begin? (Interviewers first introduce themselves and state that they are researchers from….)

INTRODUCTION (interviewer starts with a brief introduction of the study)

The study is titled "Communicating vaccine information in Nigeria". Communication is a critical component in ensuring that children are fully vaccinated and for the purpose of disease eradication and control initiatives. In Nigeria, with less than a third of the population of children fully vaccinated, issues bordering on resistance to vaccination and poor demand still persist. However, an effective communication strategy tackles most of the problems associated with low coverage, by making more people aware of the benefits of immunization; correcting false beliefs, rumors, or concerns that prevent people from getting immunized; and informing people where and when to get immunized. This study will contribute to the development of evidence-based communication strategies which will be integrated into vaccination/immunization programs. It will also serve as guidance for policy makers and programme managers on how to strengthen vaccine delivery and increase uptake in the country through the use of evidenced based communication strategies adapted for local settings. We are therefore carrying out this study to gain detailed insight into how information about vaccination is being communicated and to identify factors affecting implementation at scale.

Demographic information

1. Official title:

2. Place of work and duration of work in that position:

Interview

3. Describe how you came to be involved in the vaccination programme?

4. Please describe what you see as your current role in the vaccination programme.
   Probes:
   - How long have you worked in the area of vaccination delivery?
   - Possible functions / roles
   - Finance / financial management (all)
   - Making vaccine needs forecasts (linked to procurement) (GAVI, UNICEF, immunisation officers)

- Human resource management (all)
- Health education and promotion
- New vaccine introduction (GAVI)

5. What are the key vaccination / immunization committees in which you are involved?
    a) How does the health system at these levels (national, state and local) inform parents and public about vaccination in your setting?
    b) Do you have a name for these types of activities in your setting (e.g. social mobilization, IEC activities)?
    c) In your system who is responsible for:
        - Developing communication activities?
        - Delivering communication activities?

6. What are the communication strategies used to improve childhood vaccination uptake in your setting? (Advocacy visits, dialogue meetings, media (radio announcements, jingles on TV, information education and communication materials (posters, billboards, banners, pamphlets))
    a) What are the purposes for each intervention outlined above?
    b) Are these applicable for routine immunization (RI) or campaigns or both?
    c) What are the current programs in which communication strategies are used commonly?
    d) The content of the communication interventions? (messages)
    e) The frequency with which they are delivered and the format/s used? (how often)
    f) Who delivers the intervention?
    g) Who is communication intervention targeted to? (target audience)
    h) Are these interventions used in combination with other interventions?
    i) What vaccine preventable disease are these interventions targeting? (yellow fever, tuberculosis, Hemophilius influenza, Hepatitis B, diphtheria, pertussis)

7. To what extent are these interventions are being implemented at scale / in practice?

    Probes:
    - What has worked and what are some of the challenges encountered in relation to communicating vaccine information?
    - Find an example of a communication intervention for which scale up has been attempted, and probe what issues arose in trying to scale it up

8. Are there any laid down policy documents on how communication strategies should be carried out (at each level of government)?
    - In the EPI comprehensive multi-year plans (cMYP) (social mobilisation officers)
    - Any adaptations of the WHO 'Immunization in practice' (WHOIIP) book (should contain the operational guidelines) (UNICEF rep)

- New vaccination introduction plans (social mobilization officers, GAVI, UNICEF, WHO, SIO)
- Documents related to ‘Reaching every ward’ strategy (REW) (SIO, LIO, social mobilisation officers)
- Joints reports of National EPI, WHO and UNICEF
- EPI reviews
- GAVI proposals
- Full country evaluations (FCE) from GAVI

Request copies of these documents (where applicable):

9. Please could you describe the process from planning to implementation (if any) for some of these strategies

10. What *bottlenecks / difficulties* have you encountered/ during implementation of communication interventions from state to local government level?
    - And in what ways have they impeded? (*Probes:* disbursement of immunization materials, point of delivery, availability of health workers, allocation of jobs etc.)
11. What are the *enabling factors* for the implementation of these communication strategies? (*Probes:* political, structural, religious, etc.)

12. What is your overall impression of the National Programme on Immunization in terms of meeting its set goals? (Effective or ineffective? Give reasons)

13. Do you have any suggestions on how to improve information delivery regarding vaccination in the country?

14. What challenges to ensuring the sustainability of communicating vaccine information do you envisage, and how can these be tackled?
    - *Probe* for economic, social, religious, cultural, political challenges etc., and health system challenges, e.g., the availability of skilled personnel
