# Supplementary material for: Factors affecting the implementation of childhood vaccination communication strategies in Nigeria: a qualitative study
Source: BMC Public Health. 2017 Feb 15;17:200. doi: 10.1186/s12889-017-4020-6 (PMC5311723; doi:10.1186/s12889-017-4020-6)
Supplement: Additional file 2: — SURE Framework of key domains for the identification of factors affecting the implementation of policy options. (PDF 248 kb) [file 12889_2017_4020_MOESM2_ESM.pdf]

Additional file 2: SURE Framework of key domains for the identification of factors affecting the implementation of policy options (adapted from the SURE Framework*)

Level	Factors affecting implementation
**Recipients of care**	Knowledge and skills
	Attitudes regarding programme acceptability, appropriateness and credibility
	Motivation to change or adopt new behaviour
**Providers of care**	Knowledge and skills
	Attitudes regarding programme acceptability, appropriateness and credibility
	Motivation to change or adopt new behaviour
**Other stakeholders (including other healthcare providers, community health committees, community leaders, programme managers, donors, policymakers and opinion leaders)**	Knowledge and skills
	Attitudes regarding programme acceptability, appropriateness and credibility
	Motivation to change or adopt new behaviour
**Health system constraints**	Accessibility of care
	Financial resources
	Human resources
	Educational and training system, including recruitment and selection
	Clinical supervision, support structures and guidelines
	Internal communication
	External communication
	Allocation of authority
	Accountability
	Community participation
	Management and/or leadership
	Information systems
	Scale of private sector care
	Facilities
	Patient flow processes
	Procurement and distribution systems
	Incentives
	Bureaucracy
	Relationship with norms and standards
**Social and political constraints**	Ideology
	Governance
	Short-term thinking
	Contracts
	Legislation or regulation
	Donor policies
	Influential people
	Corruption
	Political stability and commitment

***** The SURE Collaboration: Guides for Preparing and Using Evidence- Based Policybriefs: identifying and addressing barriers to implementing policy options. Version 2.1. 2011.
